# Botulinum toxin injection combined with traditional swallowing rehabilitation improved cricopharyngeal dysfunction in neuromyelitis optica spectrum disorder: A case report

**DOI:** 10.3389/fneur.2022.939443

**Published:** 2022-07-28

**Authors:** Zitong He, Fei Zhao, Yilong Shan, Zulin Dou, Hongmei Wen

**Affiliations:** Department of Rehabilitation Medicine, The Third Affiliated Hospital, Sun Yat-sen University, Guangzhou, China

**Keywords:** neuromyelitis optica spectrum disorder, dysphagia, botulinum toxin, cricopharyngeal muscle, case report

## Abstract

Neuromyelitis optica spectrum disorder (NMOSD) is an autoimmune diseases of the central nervous system, and often influence optic nerve and medulla oblongata. Previous studies found out that brain abnormalities were not rare in these patients. Medulla oblongata (MO) was commonly involved and usually located at dorsal part. Patients who diagnosed NMOSD with MO lesions were more likely to have dysphagia. Previous reports indicated that the symptoms and signs of NMOSD patients could be controlled after immunosuppressive therapy. This patient was a 49-year-old Asian woman presented with recurrent vomiting and diagnosed NMOSD with MO involvement. However, after immunotherapy in other hospital, she still suffered from dysphagia. She then came to our department and completed videofluoroscopic swallowing study (VFSS) and high-resolution pharyngeal manometry (HRPM). Her UES was not opening with aspiration and the UES residue pressure was higher than normal range, we figured that she had cricopharyngeal (CP) dysfunction. Then the SLP gave her traditional treatment, including catheter balloon dilation. But she failed improvement after treatment for 2 weeks. Then the clinicians decided to inject botulinum toxin (BTX) into her CP muscles, which needed specific location and appropriate dosage. Her UES residue pressure decreased after three times BTX injection. During this time, her SLP adjusted the treatment strategies based on her VFSS and HRM results. Combined BTX injection with traditional treatment, she can now eat food orally without restrictions. This case report we presented can provide treatment strategies for similar patients with dysphagia.

## Background

Neuromyelitis optica (NMOSD) is an autoimmune, demyelinating, inflammatory disease affecting the central nervous system, especially affecting the optic nerve and spinal cord (1). It often occurs in female patients, and the incidence is higher in non-white ethnicity (2). Recent studies have also reported brain abnormalities were not rare in NMOSD patients. Medulla oblongata (MO), especially dorsal part, was frequently involved, with prevalence 12.8 to 91.3% (3). NMO-IgG is a specific antibody that can bind to Aquaporin 4 (AQP4) and has high sensitivity and specificity to NMO recognition (4). High AQP4 expression is generally believed to be associated with brain stem injury (5).

Previous study indicated that dysphagia frequently occurs in NMOSD with MO involvement (6–8), which can be detected through questionnaires and instrumental examination (7, 8). Also oropharyngeal swallowing disorder might lead to the pneumonia and disability (8). Some studies reported improvement in the symptoms of NMOSD (diplopia, facial palsy, dysphagia, etc.) after immunotherapy (3, 9, 10). However, few studies have reported the safety and effectiveness of these patients' swallowing process and swallowing rehabilitation. Hence, we present a case of a patient with NMOSD with MO involvement who continued to have dysphagia despite 6 months of immunotherapy.

The patient provided informed consent for the publication of this report.

## Case report

**Figure 1:**
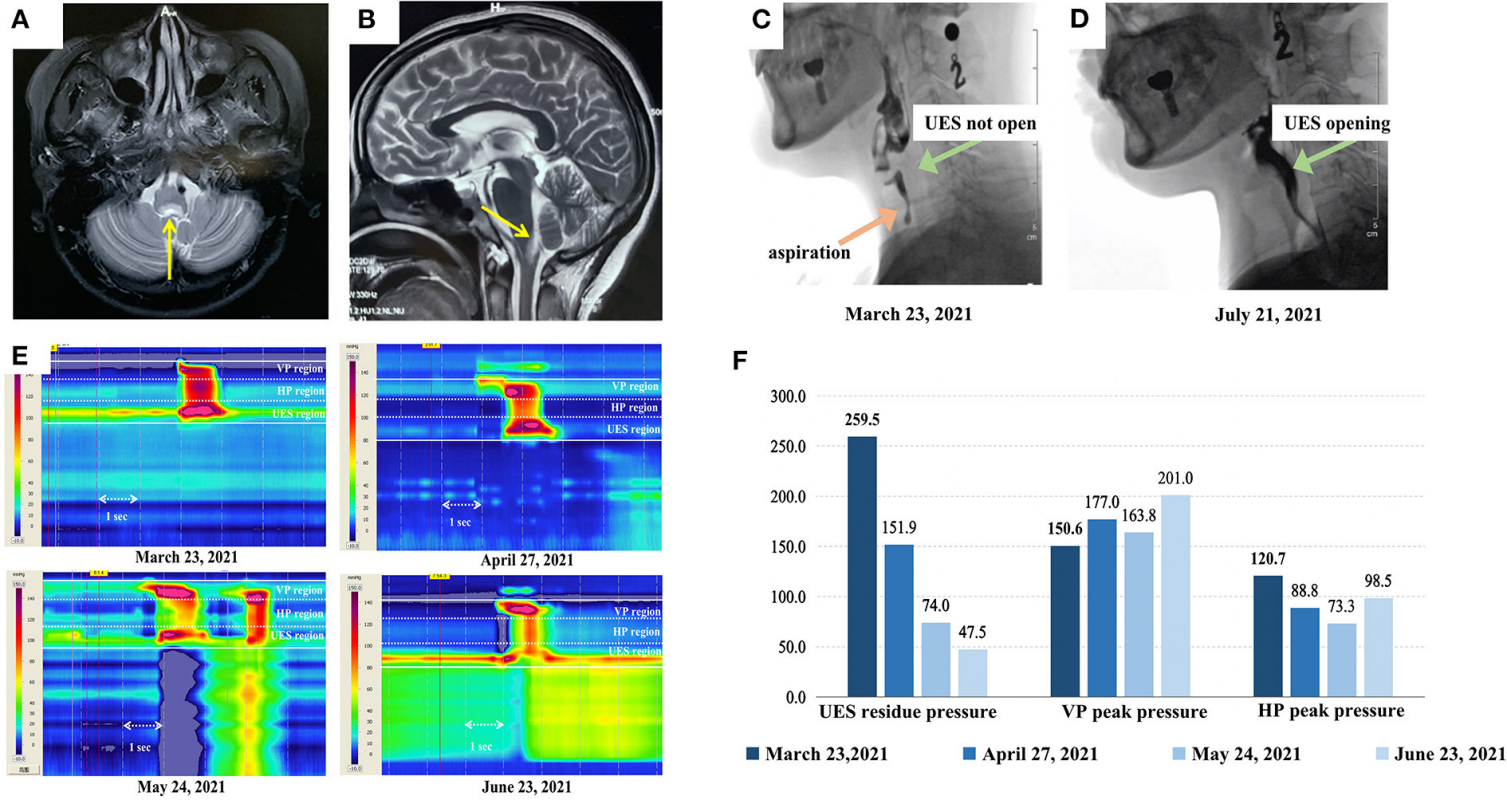
Magnetic resonance imaging (MRI) of the patient and the swallowing evaluation results **(A)** and **(B)** show the transverse and sagittal plane of the patient's brain MRI scan. The yellow arrows indicate the abnormal lesions in the medulla oblongata. **(C)** and **(D)** show the videofluoroscopic swallowing study (VFSS) imaging of this patient (captured as she ate 3 ml of extremely thick food). Her upper esophageal sphincter (UES) was completely not open with aspiration in **(C)**, while **(D)** shows that her UES was completely open with no aspiration after treatment. **(E)** is the high-resolution pharyngeal manometry (HRPM) space-time diagram of the patient (analyzed as the patient ate 3 ml of extremely thick food) and **(F)** shows the specific statistics of HRPM. The four diagrams in **(E)** and the colors from dark to light in **(F)** represent the timeline of the patients' treatment process: before the injection, and after the first, second, and third injections, respectively. In figure **(E)**, The x-axis represents time (the arrow indicates 1 s), the y-axis represents the structure from the velopharynx (VP) to the esophagus, and the color represents the pressure (mmHg) (the warmer the color, the higher the pressure). It can be observed that her UES residue pressure gradually declined. Her VP peak pressure was always in the normal range (>100 mmHg). Considering the dispersal of BTX to the hypopharynx (HP), her HP peak pressure dropped after the first and second injections. Therefore, her speech-language pathologist conducted pharyngeal balloon pressure training, then her HP peak pressure got back to normal.

A 49-year-old Asian woman presented with recurrent vomiting in September 2020. Spinal cord magnetic resonance imaging (MRI) showed no obvious abnormality of the cervical or thoracic spinal cord. Brain MRI demonstrated abnormal lesions in the medulla oblongata, bilateral hypothalamus, and white matter of the cerebral hemispheres (Figures 1A,B). Cerebrospinal fluid (CSF) analysis revealed positive oligoclonal IgG bands (OCBs). Her tests for AQP4-IgG in CSF (1:1) and serum (1:10) were positive, confirming the diagnosis of NMOSD. Her muscle strength of left upper and lower limbs were rated 5^−^ level and her right limbs were normal. Her muscular tension were normal but tendon reflex was active. Her binocular diplopia and distal vision decreased. Also, the pharyngeal reflex was disappeared. During September 2020 to the end of February 2021, she lived at local hospital and ate mycophenolate mofetil dispersible tablets 0.75 mg bid and prednisone 20 mg qd. At the end of February 2021, the overall situation has improved. Her strength of limbs and tendon reflex return back to normal level. Her pharyngeal reflex was weakened. Her binocular diplopia was alleviated, and distal vision decreased slightly. Then she visited the neurology outpatient department of our hospital on 1 April, 2021, the attending doctor adjusted the dose of prednisone to 15 mg qd and mycophenolate mofetil dispersible tablets 0.75 mg bid. She went to the neurology clinic again in June. Given the stability of the condition, the doctor reduced the dose of prednisone to 10 mg qod and mycophenolate mofetil dispersible tablets 0.75 mg bid until now. However, the dysphagia persisted even after the immunotherapy treatment. Consequently, she presented to our department in 23 March 2021 for further intervention.

Initially, a videofluoroscopic swallowing study (VFSS) indicated that she has an aspiration and her UES was not opening on swallowing 3 ml of an extremely thick bolus (Softia-S, Nutri. Co., Ltd., Japan). The first fiberoptic endoscopic evaluation of swallowing (FEES) revealed moderate residue in the epiglottis valley and piriform sinus (11). Her functional oral intake scale (FOIS) score was 1 (12). On high-resolution pharyngeal manometry (HRPM), the upper esophageal sphincter (UES) residue pressure was 259.5 mmHg when she swallowed 3 ml of an extremely thick bolus. The normal reference value for UES residual pressure is <12 mmHg (13–15), this patients' UES residue pressure was higher than the normal range. The VFSS and HRPM results represented that she had cricopharyngeal muscle (CPM) dysfunction. Hence, the speech-language pathologist (SLP) treated her with catheter balloon dilation. However, her swallowing function failed to improve after 2 weeks of training. Since the botulinum toxin (BTX) relieves muscle tone, the clinician decided to inject her CPM with 50 U of onabotulinum toxin A (Botox®, Irvine, CA, USA), diluted to 100 U/ml with normal saline, under the combined guidance of a catheter balloon, ultrasound, and electromyography (BECURE).

After the first injection, her UES residue pressure was still higher than normal (151.9 mmHg). Considering that the dosage was insufficient, another 50 U of BTX was injected into the CPM (16). Her swallowing dysfunction persisted despite 2 weeks of training. Fifteen days after the second injection, her UES residue pressure remained high (74.0 mmHg) and she could not eat. The clinicians deemed it safe and necessary to administer the third dose of 50 U of BTX into the CPM. Sixteen days after the third injection, the patient's UES residue pressure further decreased to 47.5 mmHg. And the VFSS results showed that her UES were opening without aspiration (Figure 1D). She was able to eat 100 ml of extremely thick food and spit out 20 ml. At the 6- and 9-month follow-ups after the last injection, she could consume an unlimited variety of foods orally.

## Discussion

In a study that compare different clinical features of NMOSD with and without MO involvement indicated that: dysphagia, headache, dizziness, nystagmus, dysphonia, intractable hiccup nausea, dyskinesia, and neuropathic pain are more common in patients with MO involvement. Meanwhile, MO involvement often leads to a high recurrent rate and poor prognosis (3). Milewska et al. (7) reported that 37.5% of the 72 multiple sclerosis and Devic's syndrome patients had dysphagia, of which pharyngeal dysphagia (repeated swallowing, dysphagia, coughing, feeling of food stuck in the throat) was much more common. In our study, the patient presented with severe residue in the epiglottis valley and the piriform sinus in VFSS (Figure 1C) and high UES residue pressure in HRM (Figures 1E,F), which indicated that her problem concentrated on pharyngeal stage. The patient's symptoms were consistent with previous studies. Cousins et al. (9) and Li et al. (10) reported that the symptoms and signs of dysphagia were controlled by immunosuppressive therapy in patients with NMOSD. Also in Li's case (10), the patient's symptoms did not recur after 15 months. Though the patient of our study started using immunotherapy since September 2020, she still suffered from dysphagia. After the combination of BTX injection and traditional therapy, she could eat orally. Furthermore, the evaluation of our case was comprehensive. Previous studies using FEES and questionnaires to evaluate NMOSD patients' dysphagia (7, 8). Except from FEES and subjective evaluation, we added VFSS and HRPM. VFSS clearly shown the safety and effectiveness of swallowing, while HRPM measured the muscle pressure quantitatively. With a comprehensive evaluation, we could confirm the CP dysfunction of this patient and determine what to do next.

Catheter balloon dilation is generally used in patients with CPM dysfunction. However, some patients show no improvement in swallowing even after balloon training. For these patients, BTX injection into the CPM may be considered. The CPM is a C-type skeletal muscle with a length of 1–2 cm. Precise injection is necessary for safety and effectiveness. Our team have applied this innovative technique to more than 20 patients and achieved good outcomes (13). Beside the location, the dosage of BTX is also important. Based on previous literature, the dosage of BTX was often decided by the clinician and varied from 4U to 100U (16–18), and the average dose was 39 ± 19 units (17). It was at minimum spread degree when diluted the BTX to 50-100 U/ml with normal saline (18). According to Ahsan (19), when the patients' UES residue pressure were 30 mmHg and 40 mmHg, they injected 60 U. Our team have injected 30–100 U to 21 patients with no adverse prognosis (13). The single dose was 50 U usually. Consider the above information comprehensively, we decided to inject 50 U diluted in 0.5 ml normal saline to this patient. The main considerations were to reduce side effects and have effects.

The lesions in the medullary swallowing center may be the cause of increased UES residue pressure in NMOSD patients with postrema syndrome. Three factors may affect the UES opening (20): (1). oncoming swallowed bolus-generated pressure (A); (2). anterior laryngeal movement and pharyngeal shortening-generated pressure (B); (3). UES relaxation and compliance (C). Patients can eat when A + B ≥ C. This complex series of movements is considered to be controlled by medullary swallowing center, which is called swallowing central pattern generators (CPG), especially the neucleus of the solitary tract (NTS) and other medullary or supra-medullary structure (21–24). The lesions in the medullary structure can lead to the pressure of UES, which should be decreased when swallowing, increased (21). According to the literature reported, NMOSD with MO involvement was more likely to have dysphagia (6–9). Hence, with the MO involvement may influence the CPG and NTS, and then lead to the higher UES residue pressure.

Although the UES residue pressure reduced after the first and second injections, the velopharyngeal and hypopharyngeal peak pressures remained lower than the UES residue pressure (Figure 1F), and the patient was unable to eat. Three BTX injections have rarely been reported in previous literature (16). However, since our patient's UES residue pressure remained high, a third injection was necessary. Although BTX helped in lowering the UES residue pressure, the patient's hypopharynx peak pressure dropped after the first and second injections, which might have been caused by the dispersal of BTX to the hypopharynx. Therefore, apart from the basic training, the SLP also added pharyngeal pressure training with the assistance of catheter balloon dilatation in the pharyngeal region. Per the evaluation results of VFSS and HRM, her treatment plan was changed. After all the treatment, her hypopharynx peak pressure returned to normal (Figure 1F). Also, the coordination of swallowing and neuroplasticity might be affected by catheter balloon dilation and oral intake training. Hence, the swallowing function can be maintained.

Previous studies found out that NMOSD patients with MO involvement tend to have a higher recurrence rate. According to our follow-up, the patient is currently taking a small dose of immunodrug maintenance (prednisone 10 mg qod and mofetil dispersible tablets 0.75 mg bid). She still ate orally without restriction after 1 year's treatment. We will continue to follow the patient's prognosis in future.

## Conclusion

BTX injection into the cricopharyngeal muscle, along with traditional treatment, could help improve the swallowing function in patients with NMOSD with MO involvement. This case report provides a novel treatment strategy for such patients.

## Data availability statement

The original contributions presented in the study are included in the article/supplementary material, further inquiries can be directed to the corresponding authors.

## Author contributions

ZH and FZ completed the swallowing evaluation and treatment for the patient in this case report, collected the data of this case report, completed the chart drawing, and completed the preliminary draft. YS helped revise the draft and completed the clinical examination of patients as a doctor. ZD and HW guided the operation of the whole project, revised the draft, and gave the fund. All authors contributed to the article and approved the submitted version.

## Funding

This work was supported by the [National Natural Science Foundation of China #1] under Grant [Number 81972159] and [Science and technology program of Guangzhou #2] under Grant [Number 20200703000 1].

The authors declare that the research was conducted in the absence of any commercial or financial relationships that could be construed as a potential conflict of interest.

## Publisher's note

All claims expressed in this article are solely those of the authors and do not necessarily represent those of their affiliated organizations, or those of the publisher, the editors and the reviewers. Any product that may be evaluated in this article, or claim that may be made by its manufacturer, is not guaranteed or endorsed by the publisher.
